# UIBVFEDPlus-Light: Virtual facial expression dataset with lighting

**DOI:** 10.1371/journal.pone.0287006

**Published:** 2023-09-29

**Authors:** Miquel Mascaró-Oliver, Esperança Amengual-Alcover, Maria Francesca Roig-Maimó, Ramon Mas-Sansó

**Affiliations:** Department of Mathematics and Computer Science, University of the Balearic Islands, Palma de Mallorca, Spain; Universiti Tunku Abdul Rahman, MALAYSIA

## Abstract

It is well-known that lighting conditions have an important influence on the automatic recognition of human expressions. Although the impact of lighting on the perception of emotions has been studied in different works, databases of facial expressions do not consider intentional lighting. In this work, a new database of facial expressions performed by virtual characters with four different lighting configurations is presented. This database, named UIBVFEDPlus-Light, is an extension of the previously published UIBVFED virtual facial expression dataset. It includes 100 characters, four lighting configurations and a software application that allows one to interactively visualize the expressions, and manage their intensity and lighting condition. Also, an experience of use is described to show how this work can raise new challenges to facial expression and emotion recognition techniques under usual lighting environments. Thus, opening new study perspectives in this area.

## Introduction

Facial Emotion Recognition (FER) is a research subject that sustains both an interest and a research challenge in the field of computer vision. Lighting conditions play an important role in all visual art expressions such as painting, photography, theater, film and Computer Graphics (CG) art. In particular, the lighting of virtual characters is a fundamental tool to nuance and accentuate facial expressions so that the message sent to the viewer is the one desired by the artist.

Current facial emotions databases do not consider the lighting of the characters as a characteristic. On the contrary, it is usually desired that the database be independent of lighting. However, it has been shown that the recognition of facial expressions in 3D improves as illumination increases. Therefore, recommendations of optimal lighting levels based on facial expression recognition can be a good contribution for the development of lighting standards [1].

In this work, a new database of facial expressions played by virtual characters with different configurations of lighting is introduced. The introduction of lighting in the database should allow researchers to study the effects of contrast of the key and the fill lights (KTFR -key-to-fill ratio) [2], as well as the main light positioning effects.

We want to emphasize that the dataset is the primary contribution of this work. It aims to give researchers access to data they may use to support their research and generate new knowledge. In particular, to study the effect of lighting conditions in the fields of facial expression and emotion recognition. In order to validate its usefulness and illustrate how the new dataset can open new research perspectives to facial expression and emotion recognition techniques under usual lighting environments, an experience using it is described.

## Related work

Facial expression databases can be classified into two large groups. The first group are databases generated in a controlled laboratory, like the Karolinska Directed Emotional Faces (KDEF) [3] or Extended Cohn-Kanade (CK+) [4]. The second group are databases generated in an uncontrolled environment, such as the Static Facial Expression in the Wild (SFEW) [5] or AffectNet [6]. The latter lists the main existing facial expression databases and provides a summary of the characteristics of each one. It includes the following information:

- A description of the database pointing out if it is composed by images or video sequences, the camera position (e.g., frontal, side, or stereo), the origin of the images (e.g., web, YouTube), facial points, EEG and the annotation type (manually or automatically).
- The number of subjects, which varies between 4 to 450.000.
- Its condition: controlled (posed or spontaneous) or in the wild.
- Affect modeling: number of AUs; 7 emotion categories; and valence and arousal.

Among all the facial expression databases in controlled environments only one of them, Multi-PIE [7], refers to lighting as a distinctive aspect beyond the frontal light usually considered in the character’s lighting. The Multi-PIE database uses 18 different lightings with 15 distinct cameras. Lighting is produced by flashes from the cameras plus three additional flashes added to the characters. This specific configuration is designed to provide a database with information from enough points of view and lighting to guarantee that they do not affect facial expression recognition tasks.

The impact of lighting in the perception of emotions has been studied mainly by Wisessing et al. in different works. In [2] the authors analyze the common lighting techniques to formally determine the perception of lighting and shading for animated virtual characters. They use typical lighting concepts from cinematography and photography. They refer to the three-point lighting system, consisting of three light sources: a key-light, or the main light source illuminating the scene; a fill-light that brightens up the shadow cast by the key light; and a backlight, or rim light, that separates the background from the character. Additionally, in their work, they also consider two other types of lighting—the low contrast light and the high contrast light (in terms of the relation between the key-light and the fill-light)-, and two possible lighting directions -from above and from below-. Light coming from above or “motivated light” is a natural direction light, such as the sun or a ceiling lamp. Lighting coming from below, or “unmotivated light”, is used to add dramatic effects. They also consider the case of no visible directional light or “no light”. In a subsequent work [8], the same authors explore the importance of brightness and shadow intensity on the perception of appeal, recognition, and intensity of emotions for CG characters and animation. As the authors state, the main findings of this work are the following:

- Despite the emotions portrayed, the higher the brightness the more appeal the characters have.
- Some emotion’s intensity can be altered by changing the brightness. Happy and sad emotions have contrary behaviors: bright conditions intensify happiness and dark conditions intensify sadness.
- Lighting conditions do not alter the perception of anger and fear.
- The appeal of realistic characters is not altered by dark shadows, while cartoon characters’ appeal is diminished.

These findings are extracted from an experiment with fifteen participants that saw four characters with registered audios (neutral) and animations. The authors use the three-point lighting and conclude that the key-light direction does not affect the perception of the intensity of the emotion.

In [9] the author also investigates the effects produced by lighting in 3D animated scenes on the emotion and perception of the viewer. In this work emotions are measured by performing an experiment with 72 participants. Results show that a significant emotional effects were only found in low-key lighting compared to no lighting. Moreover, they were only present in negative affect and negative emotions. Interaction between the color of light and lighting style was present for positive affect, negative affect, and basic positive emotion scales.

Even though the impact of lighting in the perception of emotions seems to be proven, to the best of our knowledge, there is no database of facial expressions that considers intentional lighting.

### The UIBVFED database

UIBVFED is a virtual facial expression database [10]. This is the first database made up of synthetic avatars that categorize up to 32 facial expressions. The UIBVFED dataset contains 640 facial images that recreate 32 facial expressions played from 20 virtual characters. The database has 10 men and 10 women, with an age ranging from 20 to 80, from different ethnicities. Expressions are classified based on the six universal emotions (Anger, Disgust, Fear, Joy, Sadness, and Surprise) according to Faigin’s classification [11]. Characters have a 2048x2048 texture resolution assigned to the color channel, specular channel and bump mapping channel. Textures give the characters a realistic appearance showing brightness and wrinkles. The database was generated in the Unity 3D development environment. The scene is neutral, uses a white background and has three-point classical lighting with minimum contrast. More specifically, the lighting ratio between the key-light and the fill-light is 1.5:1. In addition to Faigin’s expression classification, the database provides the equivalence of the Facial Action Coding System (FACS) [12] with information about the position of the 51 facial landmarks in the 3D space to facilitate expression recognition. This is because the images of the facial expressions in the dataset were generated following the guidelines of the Facial Action Coding System (FACS); meaning that the deformations that were applied to the 3D models have a direct correspondence with the Action Units (AUs) that are associated with each expression. This procedure ensures an objective labeling of all the images.

The dataset is provided together with an interactive application, the UIBVFED application GUI, that allows the users to activate and control the expression intensity of the characters from different points of view.

Synthetic datasets have shown to be a good replacement for real-image datasets since they achieve recognition rates that are comparable to the genuine ones [13, 14]. In particular, the UIBVFED dataset has been used in several FER and Emotion Recognition (ER) studies [15, 16]. However, despite the large number of facial expressions categorized, this original version of the dataset has a limited number of characters, i.e., a limited number of samples per category. Therefore, regardless of light conditions, it is also necessary to extend the number of characters in the database.

## The UIBVFEDPlus-Light database

The database presented in this article is an extension of the UIBVFED database [10]. This new database, named UIBVFEDPlus-Light, includes 100 characters and four lighting configurations. The software application that allows to interactively visualize the expressions has also been extended with a new functionality to control lighting options. In the following subsections these three aspects are exposed in more detail.

### UIBVFEDPlus-Light database generation process

UIBVFEDPlus-Light has 100 virtual characters, compared with the 20 of UIBVFED. These characters were developed with the online interactive tool Autodesk Character Generator [17]. This tool allows to handle facial characteristics—such as the face oval, the eyes or the ears—from 92 basic characters (46 males and 46 females).

As in UIBVFED, the blendshapes system allows characters to reproduce the 32 facial expressions classified according to Faigin [11]. These expressions were grouped into the 6 universal expressions plus the neutral expression. Muscle deformers to define the expressions were used. A spreadsheet with the values of each deformer is included as supplementary material.

**Fig 1:**
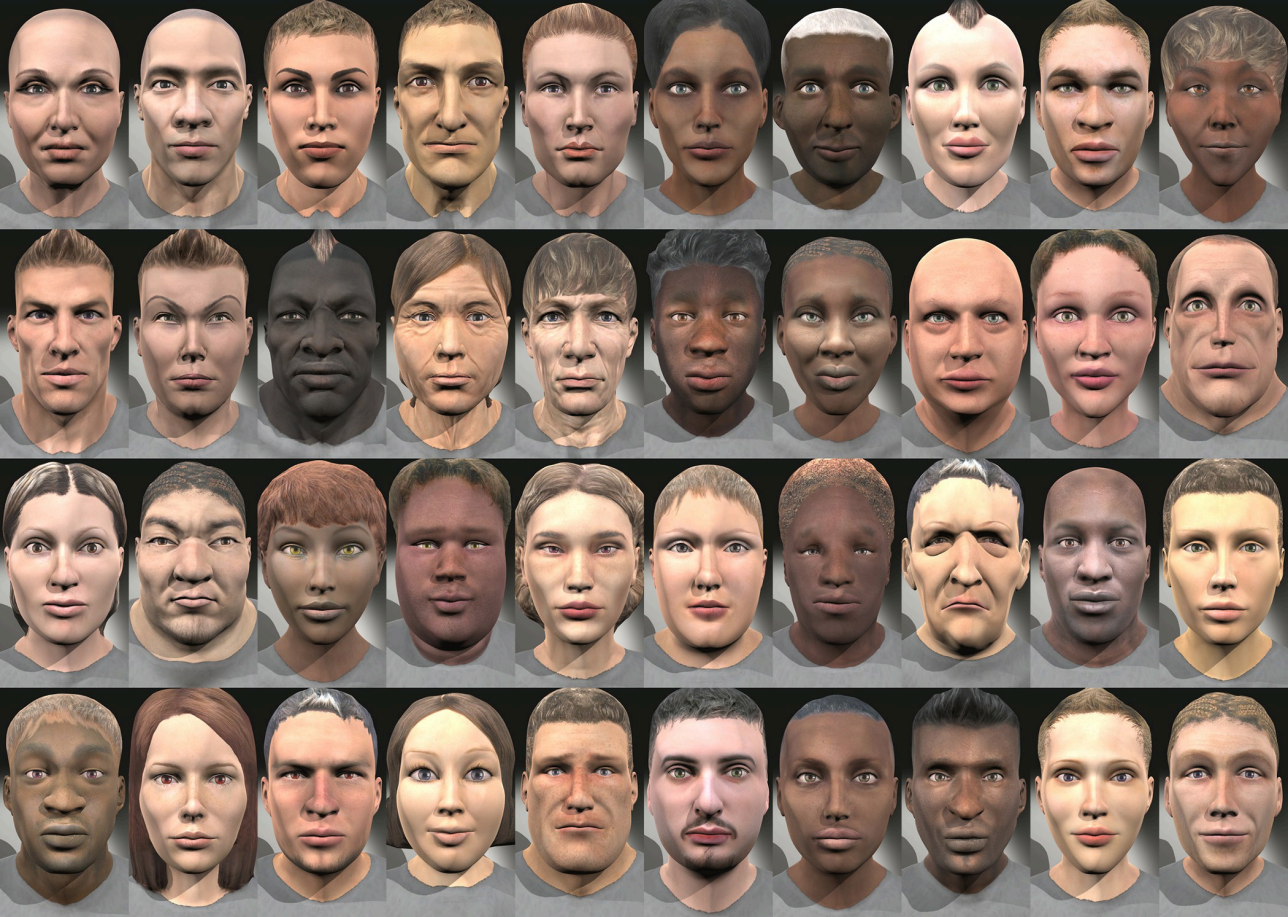
Example of UIBVFEDPlus-Light characters.

The database is composed of 50 women and 50 men from different ages and ethnicities. Fig 1 shows an example of 40 characters of the database reproducing the neutral expression with a standard light with no contrast. The age range of the characters is 20 to 80 years. The ethnic type is also defined by the morphology of the geometry and especially by the color texture applied. With the Autodesk Character Generator it is possible to select 96 textures for different skin tones, 48 for women and 48 for men. These skin tones cover all ethnic groups and allow to add variations of makeup and facial hair.

**Fig 2:**
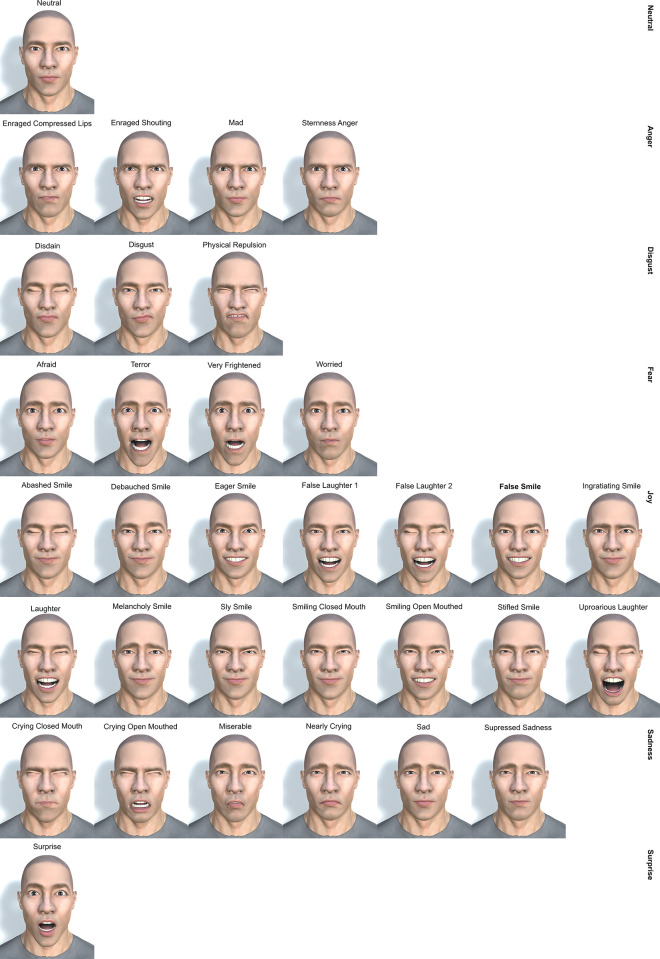
Char002 reproducing the 33 different expressions.

Fig 2 shows a specific character, Char002, reproducing 32 facial expressions -plus the neutral facial expression- divided into: 1 neutral, 4 anger, 3 disgust, 4 fear, 14 joy, 6 sadness, and 1 surprise. Therefore, for 100 characters the total sample is: 100 neutral, 400 anger, 300 disgust, 400 fear, 1400 joy, 600 sadness, and 100 surprise.

### Introducing lighting in UIBVFEDPlus

In UIBVFEDPlus-Light the scene has a white background with the shadow of the characters that depends on the lighting at a particular time.

To introduce lighting in the database the eight-light system described by Alton [18] was simplified resulting in four possible lightings with a common three-light system:

- Key-Light: the main light that defines the lighting and gives its meaning;
- Fill-Light: ensures that no area of the image is without light and softs the shadows of the Key light;
- Back-Light: also named Separated Light or Rim light, that outlines the character and separates it from the background.

Lighting variations were created in the context of a scene using the Unity 3D software. All lights are white and can be located in two different positions for the Key-Light: one light coming from above and a second one coming from below the face. The other two lightings were introduced with variations of the KTFR (key-to-fill ratio). Values used in “no contrast” lighting are 1.5 for both the Key-Light and the Back-Light. For contrast lighting values are 2 for the Key-Light and 0.2 for the other two (Fill-Light and Back-Light). These values correspond to light intensity in Unity that increases light colors allowing values from 0 to 8. The default value is 1. Higher values provide brightness and saturation.

**Fig 3:**
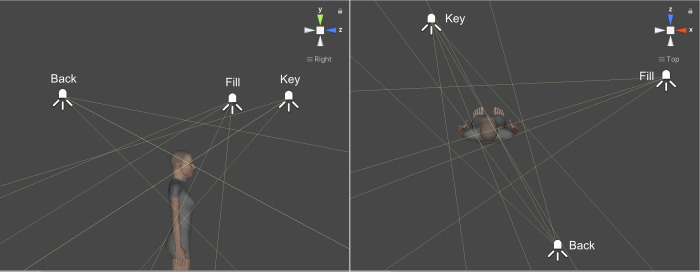
High Key-Light lighting configuration (standard).

**Fig 4:**
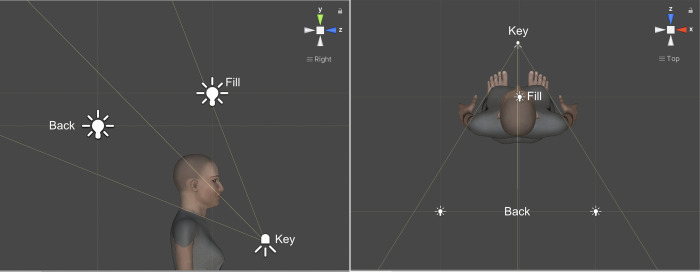
Low Key-Light lighting configuration.

Figs 3 and 4 show two lighting configurations depending on the Key-Light position. The lights used are Point Light and Spot Light according to the Unity type property. Both are located at a point in the scene and create shadows. The first emits light in all directions equally whereas the second emits light in a cone shape.

**Fig 5:**
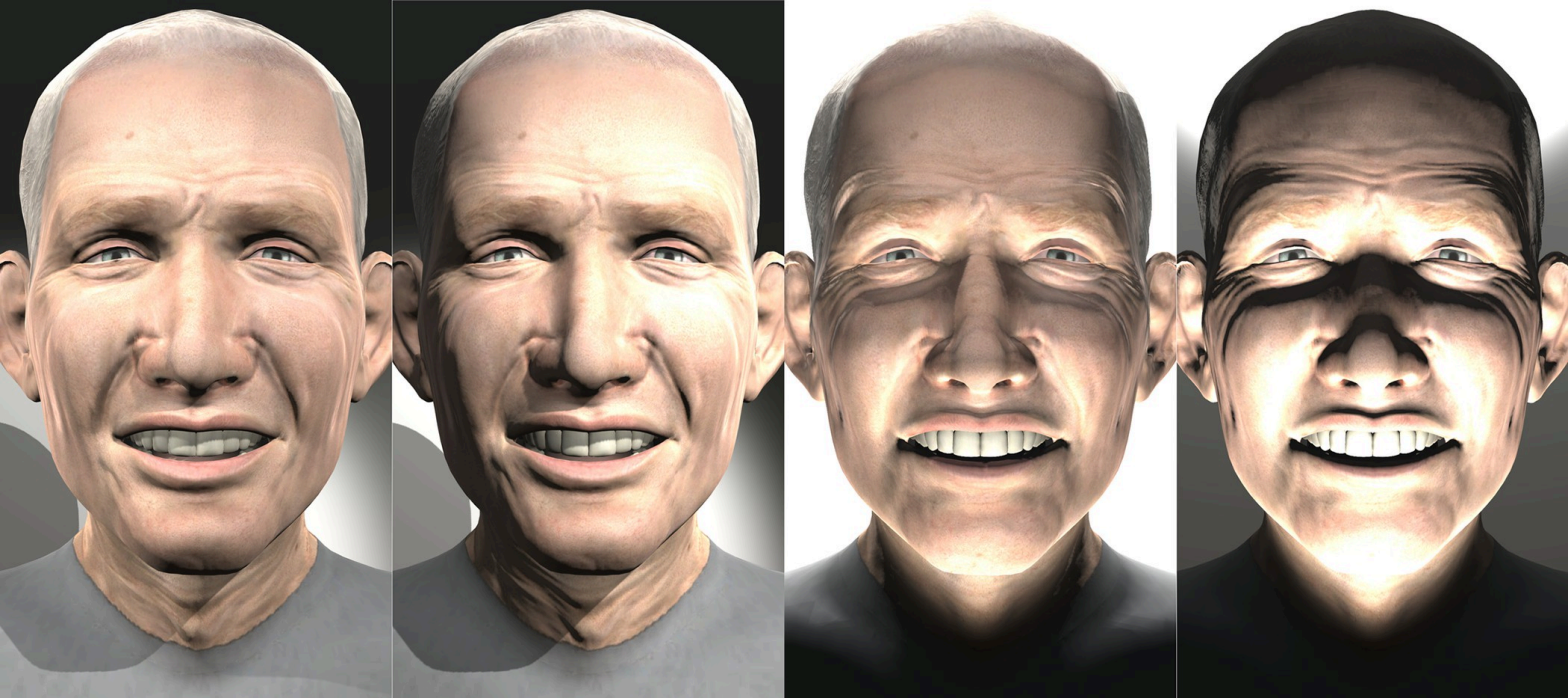
Char100 Smile Open Mouthed expression with 4 lighting configurations.

Fig 5 shows the four lighting configurations applied to the same character, Char100, with the same expression (Smile Open Mouthed). The four configurations are, from left to right: *from above without contrast*, *from above with contrast*, *from below without contrast*, and *from below with contrast*.

In all, UIBVFEDPlus-Light is composed of 13200 images with a pixel resolution of 1080x1920. That is, 3300 images for each type of lighting.

### Software application new functionality

As mentioned before, UIBVFED includes a software application that allows users to interactively visualize the expressions. This tool has been extended to visualize the expressions and manage them with the functionality defined in UIBVFEDPlus-Light. The application can be executed in a WebGL environment. It is available for any browser at http://ugivia.uib.es/uibvfed-plus-light/.

**Fig 6:**
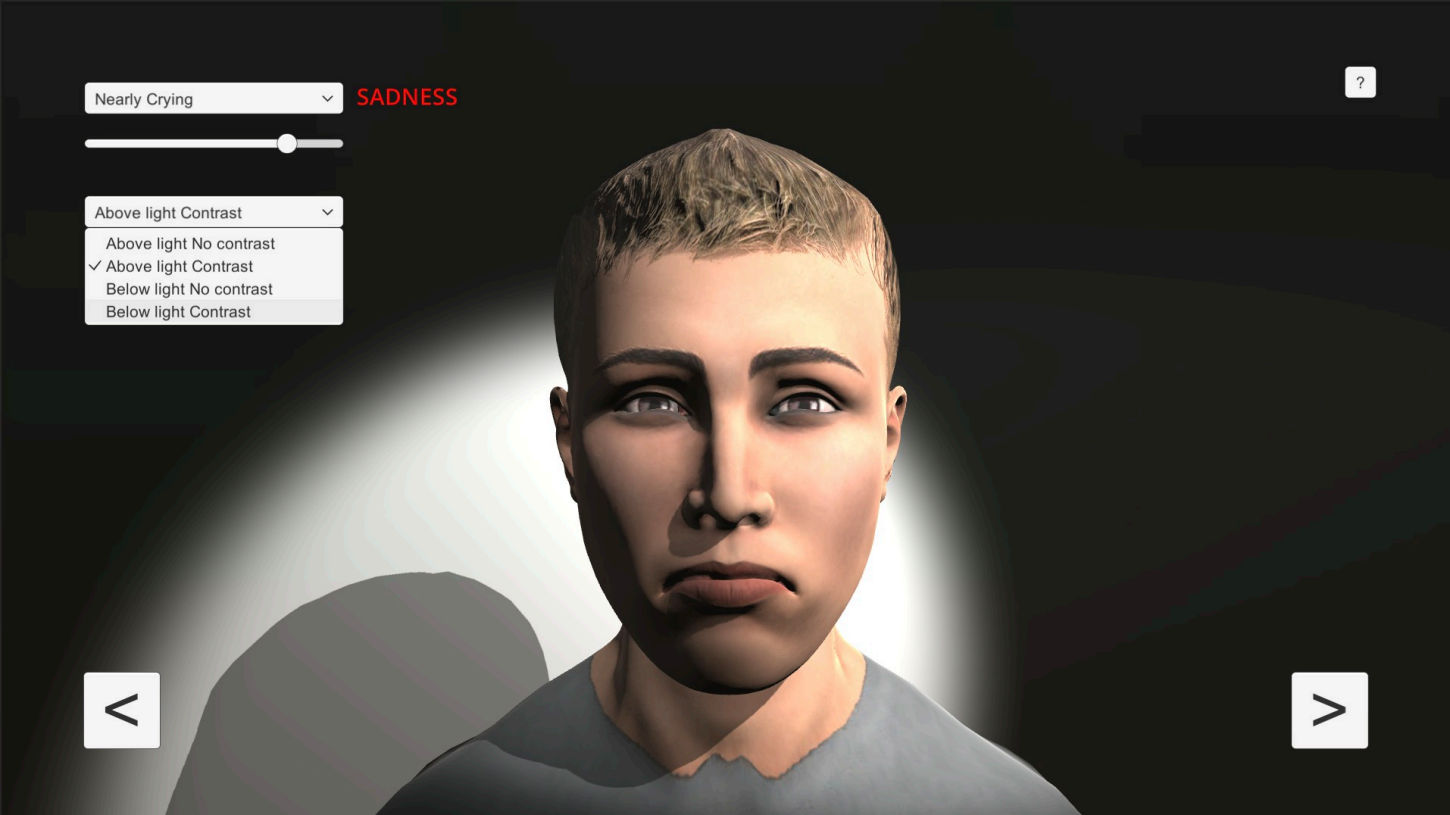
UIBVFEDPlus-Light application GUI.

Fig 6 shows the application graphical user interface with the new features adding the lighting functionality option.

Fig 6 shows the following functionalities:

- A dropdown menu to select a facial expression with a slider that allows to adjust the expression intensity.
- A dropdown menu to select the lighting type.
- Two arrow buttons to select among 30 different characters.
- The help menu.

The arrow keys allow users to rotate the character as well as zoom in and zoom out.

#### Facial landmarks

**Fig 7:**
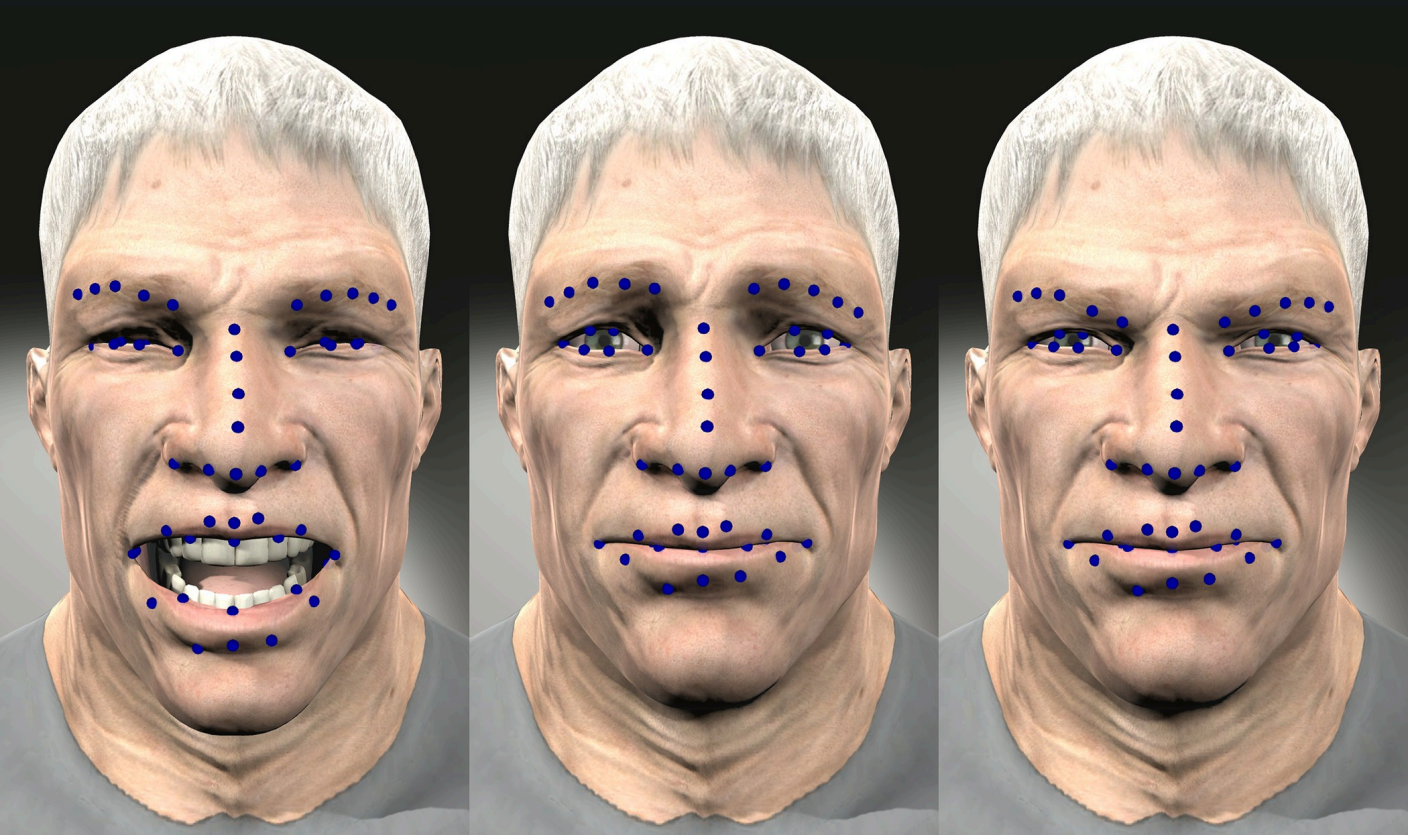
Char074 Laughter, Melancholy Smile and Sly Smile expressions with landmarks.

UIBVFEDPlus-Light includes an extension regarding the position of the 51 facial landmarks to facilitate expression recognition. The new landmarks model is similar to the one proposed by Sagonas [19] shown in Fig 7.

These landmarks were generated by selecting the specific vertices from the character’s polygon meshes. This allows to have the spatial coordinates of each of the 51 points for all characters and for all expressions.

The database provides 3300 images that correspond to the lighting configuration *from above without contrast* with the landmarks displayed as blue points as shown in Fig 7. In addition, for each character a file contains 3D vertex information with the name of the expression and the values of the 51 points (5 for the right eyebrow, 5 for the left eyebrow, 9 for the nose, 6 for the right eye, 6 for the left eye, 12 for the mouth, and 8 inside of mouth). The values of these points are 3D spatial coordinates in Unity. The character is located in the origin of the coordinate system. All characters are the same height.

## An experience using the UIBVFEDPlus-Light dataset

In a simple Convolutional Neural Network (CNN) previously trained to recognize the six universal emotions–plus the neutral emotion–with the UIBVFED dataset (the original facial expression dataset with only 20 synthetic avatars) [20], an overall accuracy of 0.88 was obtained.

To test the possibilities of the new UIBVFEDPlus-Light dataset, the same simple CNN model was trained and tested with the facial expression images generated with the lighting configuration *from above without contrast*, obtaining an overall accuracy of 0.93 (more detailed information about the training and testing followed procedure can be found in Castillo Torres et al. [21]).

**Fig 8:**
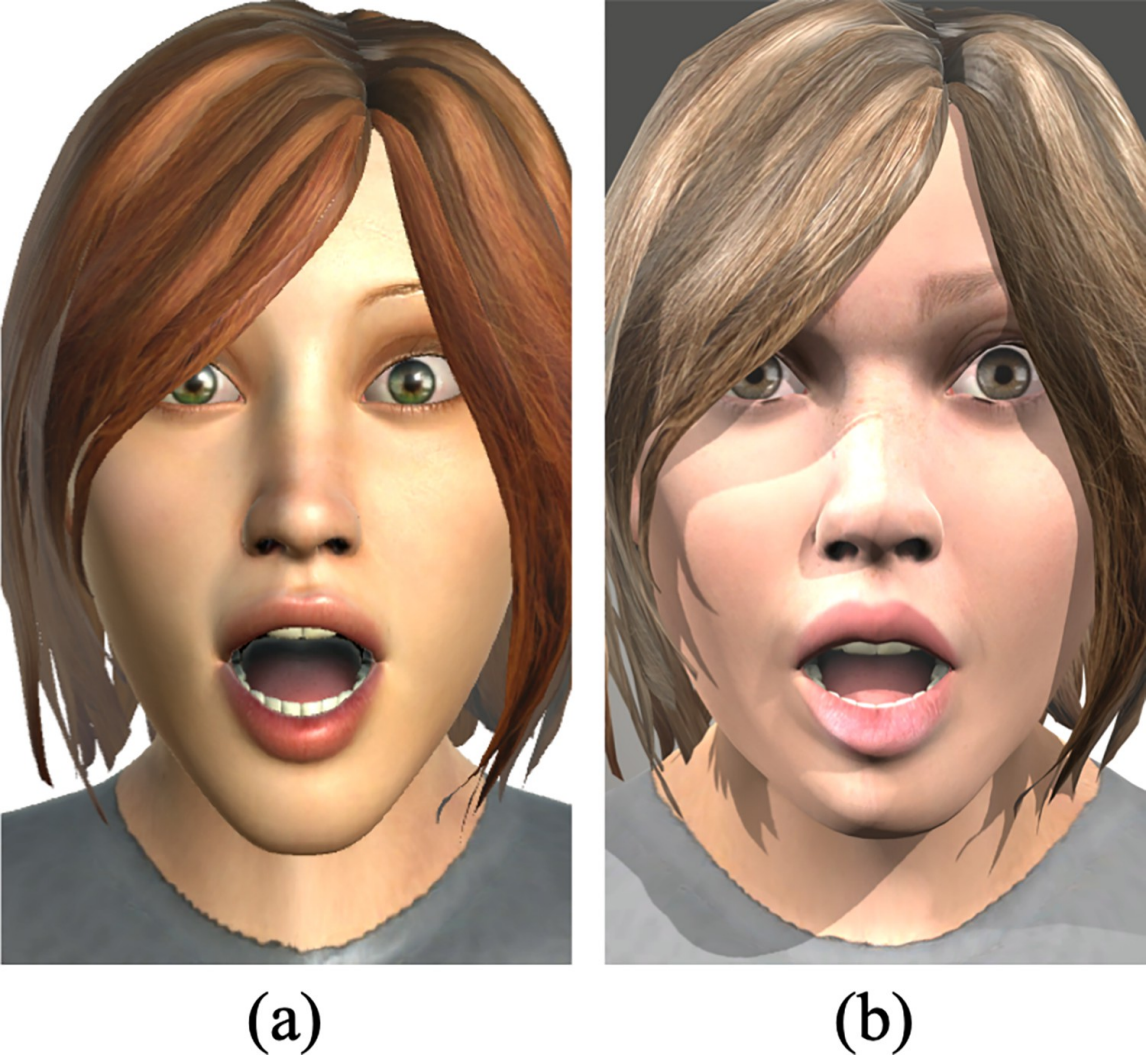
Example of the character Char041 reproducing the *surprise* facial expressions (associated to the emotion of surprise) in UIBVFED and UIBVFEDPlus-Light.

Fig 8 shows the same synthetic avatar performing the same facial expressions in the (a) UIBVFED original dataset and in the (b) UIBVFEDPlus-Light dataset with the lighting configuration *from above without contrast*. As seen in Fig 8, the resolution of the images and the realism of the avatars have both been improved. However, the dataset’s increased image count (from 660 to 3300 images) may be the primary factor in the improved overall accuracy obtained by the model (an improvement of 5.7%).

**Fig 9:**
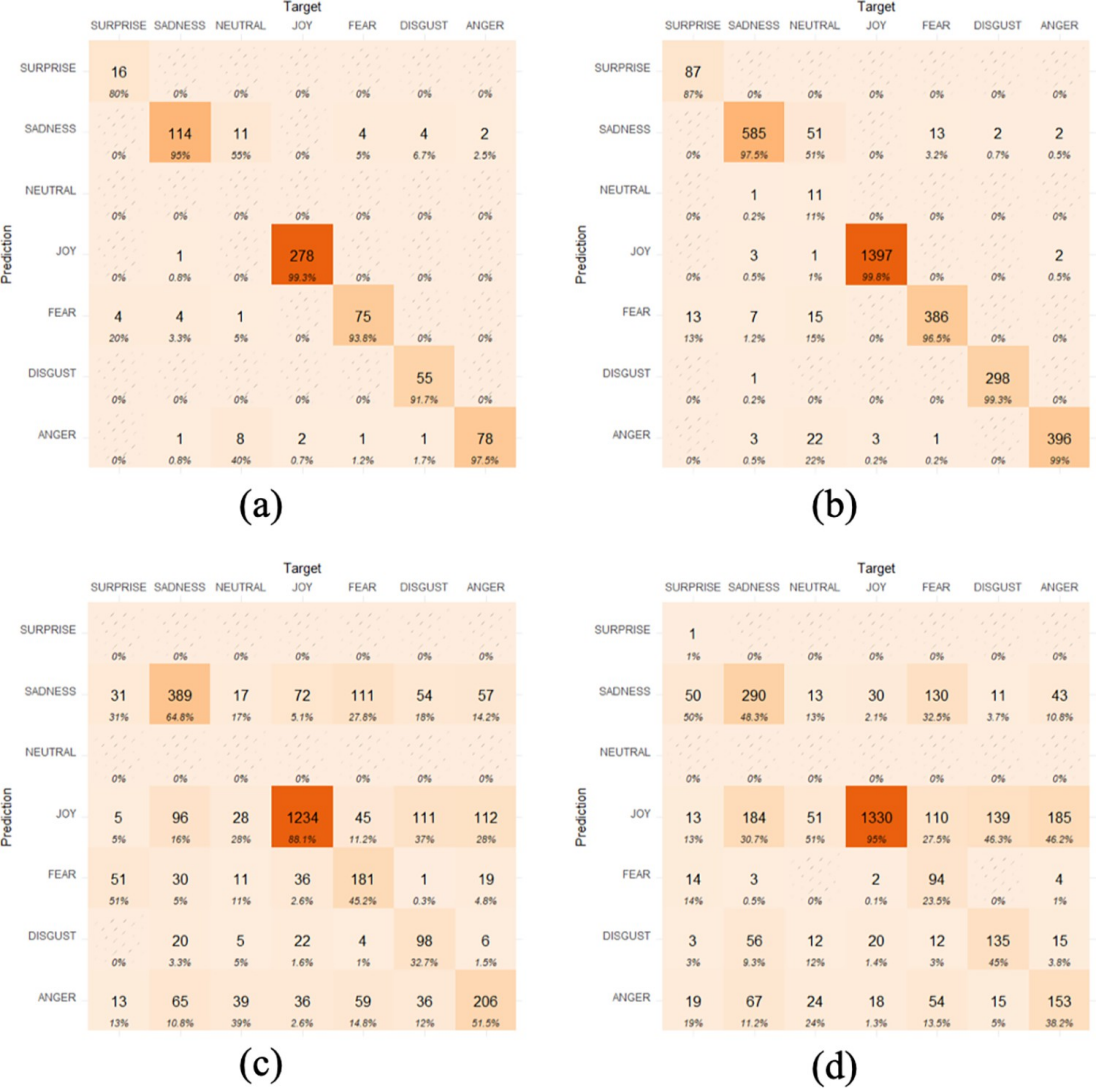
Confusion matrices of the emotions’ classification performed by the CNN model.

The CNN model trained with the images with the lighting configuration *from above without contrast* has also been tested with all the remaining lighting configurations. The confusion matrices from each testing situation are displayed in Fig 9: (a) *from above without contrast*, (b) *from above with contrast*, (c) *from below without contrast*, and (d) *from below with contrast*. Darker colors correspond to higher accuracy.

**Fig 10:**
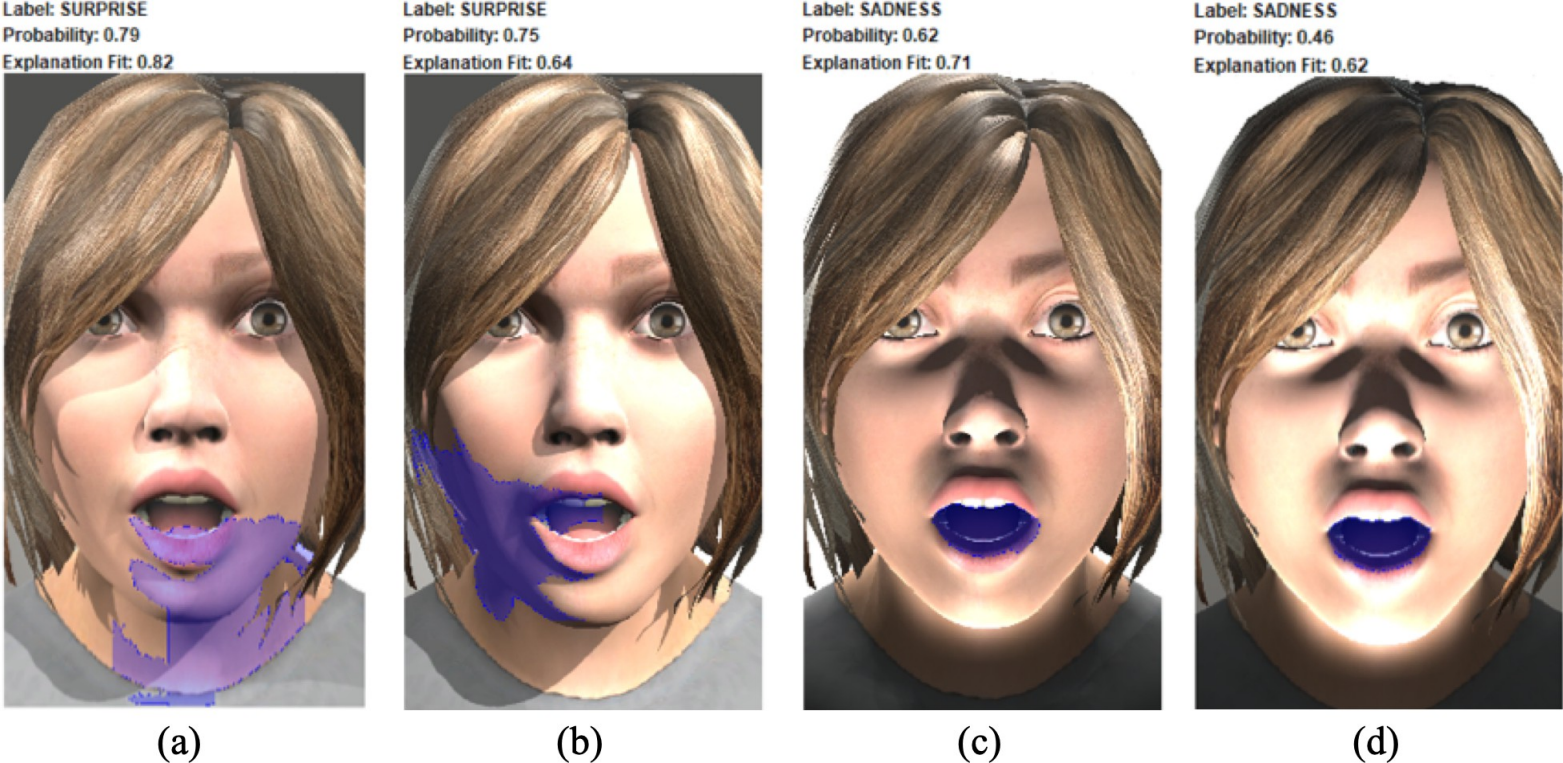
Results of applying LIME to the classifications obtained with the CNN model.

The resulting class classifications shown in Fig 9 demonstrate the impact of lighting conditions on the classifier’s ability to recognize emotions. As a result, additional research should be done to thoroughly examine the impact of lighting. Such discoveries offer new challenges in the fields of emotion and facial expression recognition. To mention just an example, it is remarkable the impact of lighting on the recognition of the surprise emotion: from an accuracy of around 80% with the lighting conditions *from above*, to a dramatic 0–1% when the lighting comes *from below*. To try to understand the behavior of the CNN model and to try to explain the classifications obtained, the Local Interpretable Model-agnostic Explanations (LIME) method [22] was applied. LIME is a method of Explainable Artificial Intelligence (XAI) that emphasizes the parts of an image that are important for a certain classification in models that deal with images. XAI was established in order to aid in the interpretation of the findings and provide a logical justification for the judgments made to the prediction of a neural network model [23]. Fig 10 shows the results of applying LIME to the classification obtained with CNN model for the images of the character Char041 reproducing the *surprise* facial expression (associated to the emotion of surprise) with all the lighting configurations: (a) *from above without contrast*, correctly labeled as the emotion of surprise, (b) *from above with contrast*, correctly labeled as the emotion of surprise, (c) *from below without contrast*, incorrectly labeled as the emotion of sadness, and (d) *from below with contrast*, also incorrectly labeled as the emotion of sadness.

As seen in Fig 10, in all the cases, the model focuses on the facial features of the region of the mouth and chin to classify the images. For the lighting condition *from above* (see Fig 10(A) and 10(B)), it seems that the decisive feature considered to correctly classify the images as a surprise emotion is the opening of the mouth without any muscle tension (notice that the highlighted parts of the images by LIME correspond to the region where the muscles of the mouth would show the presence or absence of tension). In the cases where the lighting comes *from below* (see Fig 10(C) and 10(D)), the model focuses on the facial features of the area of the mouth; this shifting may be due to the fact that the lighting from below causes a shadow inside the mouth, darkening this area; and, therefore, standing it out. The darkening of the area inside the mouth could raise the effect of “mouth widened and stretched” characteristic of the facial expression of *crying open-mouthed* associated with a sadness emotion.

## Limitations

The UIBVFEDPlus-Light dataset presented in this work has to be seen in light of some limitations.

First, the dataset is composed of decontextualized images. The correct interpretation of a particular facial expression by a human is not only based on the facial morphology, but also on other factors such as the context, sound, body movement or other physiological characteristics of the subject who performs the expression. This poses a serious drawback for humans when interpreting expressions, particularly in categorizing into the 33 different expressions from our dataset. In this study we introduce lighting to contextualize images, thus opening a field of research in this sense.

Second, the facial expressions of the 100 characters in the dataset are generated based on deformation values which are specific for each expression. Although, according to the literature, there is a single AUs configuration for each labeled expression, in a more realistic assumption, each character could introduce intensity variations. In the proposed dataset, differences in a specific expression for different characters are only due to differences in the polygonal facial mesh of each character. This can introduce a bias when interpreting them.

Finally, the dataset has been built based on Faigin’s morphological description (33 facial expressions plus the neutral one) [11]. This reference is the standard used by graphic artists to represent facial expressions. In Faigins’s work the face is divided into zones to study how the activation of certain facial muscles allows the 33 categorized expressions. Interpretation of each expression is based on pictorial, sculptural or cinematographic works conveniently contextualized. Neither this interpretation nor our dataset are validated in any kind of experiment with humans.

Each of these limitations represents new challenges for future work as well as possible improvements of the UIBVFEDPlus-Light dataset.

## Conclusions and future work

This work is oriented to multidisciplinary areas that could benefit from facial expression virtual prototyping. It offers a 3D avatar expression dataset to the research community. The UIBVFEDPlus-Light completes the work developed with the UIBVFED database. It is the first database with virtual characters and 33 different facial expressions that considers the usual types of light in character lighting. Moreover, the increase in the number of characters alleviates overfitting in learning processes for FER. The application GUI allows users to select a facial expression, manage expression intensity and select among four lighting variations. The main contribution of this new database is that it poses new challenges to FER techniques under usual lighting environments, hence opening new study perspectives in this area. From an artistic point of view, this work can be of great help. Artists can use the UIBVFEDPlus-Light tool to understand in a precise and agile way the lighting effects on facial expression of different characters. Similar to the original UIBVFED, the new database has some limitations. All characters use the same deformation value for a specific expression. Obviously, this can be seen as an inaccuracy. In reality, the intensity of an expression for each individual depends on different psychological or physiological parameters that the system cannot reflect. However, the resemblance among characters with the same expression is mitigated by the differences among the facial geometries topology for each character. As future work it is planned to improve the character’s realism and include movement from the exploration of the work environments.

## Supporting information

S1 Text (TXT)
